# EspA Acts as a Critical Mediator of ESX1-Dependent Virulence in *Mycobacterium tuberculosis* by Affecting Bacterial Cell Wall Integrity

**DOI:** 10.1371/journal.ppat.1000957

**Published:** 2010-06-24

**Authors:** Alejandra Garces, Krishnamohan Atmakuri, Michael R. Chase, Joshua S. Woodworth, Bryan Krastins, Alissa C. Rothchild, Talia L. Ramsdell, Mary F. Lopez, Samuel M. Behar, David A. Sarracino, Sarah M. Fortune

**Affiliations:** 1 Harvard School of Public Health, Department of Immunology and Infectious Diseases, Boston, Massachusetts, United States of America; 2 Brigham and Women's Hospital, Division of Rheumatology, Boston, Massachusetts, United States of America; 3 Thermo Fisher Scientific, BRIMS Unit, Cambridge, Massachusetts, United States of America; University of Washington, United States of America

## Abstract

*Mycobacterium tuberculosis* (*Mtb*) requires the ESX1 specialized protein secretion system for virulence, for triggering cytosolic immune surveillance pathways, and for priming an optimal CD8+ T cell response. This suggests that ESX1 might act primarily by destabilizing the phagosomal membrane that surrounds the bacterium. However, identifying the primary function of the ESX1 system has been difficult because deletion of any substrate inhibits the secretion of all known substrates, thereby abolishing all ESX1 activity. Here we demonstrate that the ESX1 substrate EspA forms a disulfide bonded homodimer after secretion. By disrupting EspA disulfide bond formation, we have dissociated virulence from other known ESX1-mediated activities. Inhibition of EspA disulfide bond formation does not inhibit ESX1 secretion, ESX1-dependent stimulation of the cytosolic pattern receptors in the infected macrophage or the ability of *Mtb* to prime an adaptive immune response to ESX1 substrates. However, blocking EspA disulfide bond formation severely attenuates the ability of *Mtb* to survive and cause disease in mice. Strikingly, we show that inhibition of EspA disulfide bond formation also significantly compromises the stability of the mycobacterial cell wall, as does deletion of the ESX1 locus or individual components of the ESX1 system. Thus, we demonstrate that EspA is a major determinant of ESX1-mediated virulence independent of its function in ESX1 secretion. We propose that ESX1 and EspA play central roles in the virulence of *Mtb in vivo* because they alter the integrity of the mycobacterial cell wall.

## Author Summary

From studies of BCG, the tuberculosis vaccine, we know that *Mycobacterium tuberculosis* requires a specialized protein secretion system, ESX1, to cause disease in people. ESX1 is required for *Mtb* to co-opt the host cells in which the bacterium resides and it is thought that this explains its central role in virulence. However, other data suggests that ESX1 serves an important role in the bacterium itself, altering the organism's cell wall. It has been difficult to determine the relative significance of these ESX1-associated functions, however, because deletion of any piece of the apparatus completely abolishes all ESX1 activities. Here we use a simple approach to pinpoint the functionally significant target of one of the proteins secreted by ESX1, EspA. We mutate EspA such that the ESX1 system still secretes its substrates but the bacterium no longer causes disease. The attenuated EspA mutant has defects in its cell wall but not in its interactions with host cells *in vitro*. We propose that the ESX1 system and the proteins it secretes are important for *Mtb* to survive and cause disease in people because they act to ensure the integrity of the bacterial cell wall.

## Introduction

*Mycobacterium tuberculosis* (*Mtb*) is a devastating pathogen that causes epidemic disease and latently infects much of the world's population. However, the molecular details of its pathogenesis are poorly understood. Many lines of evidence underscore the importance of an alternative protein secretion system, ESX1, to *Mtb* survival in the macrophage and virulence in animals. The primary attenuating deletion in the vaccine strain, *Mycobacterium bovis* BCG is the loss of nine genes from the ESX1 locus [1]–[4]. Deletion of the ESX1 locus from virulent *Mtb* significantly attenuates the bacterium for growth in macrophages and animals [5]–[6]. ESX1 has been implicated in the ability of the bacterium to trigger macrophage production of IFN-β [7]–[8], activate the inflammasome [9], modulate macrophage cytokine production and signaling [5], and escape from the phagolysosome [10]–[11]. The ESX1 substrate proteins are also important targets of the adaptive immune response and are recognized by both CD4+ and CD8+ T cells in a majority of infected individuals [12].

The primary function of ESX1 activity in mediating virulence is unknown, however. There are data demonstrating that ESX1 is required for *Mtb* to damage the host cell membranes but it is less clear whether this is a direct function of the ESX1 locus. *Mtb* induces IFN-β production during macrophage infection by activation of the cytosolic pattern receptors [8], [13]. ESX1 dependent escape from phagolysosomes [10]–[11], [14] could similarly result from ESX1-mediated membrane damage. This has been hypothesized to be the direct effect of one of the ESX1 substrates, EsxA (Esat6) which has been found to be capable of forming pores in a variety of membrane systems [2], [15]–[16].

The pore-forming function of EsxA is controversial, however, in part because the ESX1 locus and EsxA are highly conserved in nonpathogenic gram positive organisms that lack obvious pore-forming ability [17]–[18]. In non-pathogenic organisms, ESX1 function has been associated with intrinsic bacterial processes including conjugative DNA transfer [19] and phage susceptibility [20] although the molecular basis for this is unclear. Interestingly, in pathogenic mycobacteria, loss of ESX1 function has also been associated with changes in colony morphology. Both *M. bovis* BCG and H37Ra, which are spontaneous mutants of virulent mycobacteria that were attenuated through loss of ESX1 function [21]–[23], were initially isolated from populations of virulent organisms because of changes in their colony morphology. When BCG was complemented with a wildtype copy of the ESX1 locus, the colony morphology reverted to that of virulent *Mtb* [3]. These data have suggested that ESX1 activity modifies *Mtb* cell wall composition although the basis for these observations is also unclear.

None of the ESX1 substrates has predicted cell wall modifying activity. In addition to EsxA, four other substrates of the ESX1 locus have been reported in *Mtb*. EsxB (Cfp10) heterodimerizes with EsxA and appears to direct its secretion [24]–[25]. ESX1 secretes a transcriptional regulator, EspR (Rv3849) [26] and two proteins of unknown function, EspA (Rv3616c) [27] and EspB (Rv3881) [28]–[29]. Identifying a unique function for any ESX1 substrate has been complicated by the fact that EsxA, EsxB, EspA and EspB require each other for secretion [27]–[28]. Thus, it has not been possible to use a loss-of-function approach to define the distinct activities of the individual substrate proteins.

In this study, we designed a novel strategy to determine whether the EspA has an independent role in virulence beyond its role in codependent secretion, using a structure-function approach to examine determinants of EspA's post-secretory activity. We demonstrate that EspA forms disulfide bonded homodimers after secretion and that abrogation of EspA disulfide bond formation does not alter protein secretion, the ability of *Mtb* to trigger the IFN-β response, or to stimulate robust CD4+ and CD8+ T cell responses. However, blocking EspA disulfide bond formation significantly attenuates the virulence of *Mtb* in animals and this attenuation correlates with a loss of cell wall integrity. Taken together, these data suggest that ESX1 is required for *Mtb* to survive and cause disease in animals in part because the full activity of at least one of its substrates, EspA, is required to maintain the structural integrity of the mycobacterial cell wall.

## Results

### Secreted EspA forms disulfide bonded homodimers

**Figure 1:**
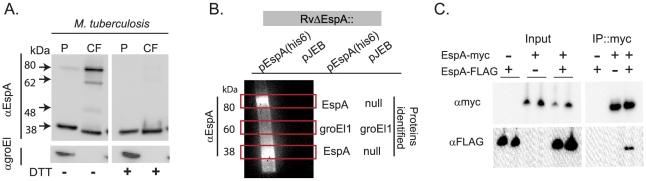
EspA forms disulfide dependent homodimers in wildtype *Mtb*. A. Whole cell pellets (P) and short term culture filtrates (CF) from wildtype *Mtb* (H37Rv) grown in N-salt media were analyzed by SDS-PAGE under nonreducing conditions and Western blot for EspA and groEl1, a lysis control. Results are representative of three independent experiments. B. Proteins were affinity purified with nickel agarose from RvΔ*espA* complemented with the indicated vectors. Purified proteins were analyzed for the presence of EspA by Western blot analysis. Regions of the corresponding gel with bands visible by Coomassie staining are indicated with boxes. Protein composition of these bands was determined by LCQ-MS/MS (38 kDa band) or LTQ-FT-MS/MS (60 and 80 kDa bands). C. Immunoprecipitation of the EspA homodimer from *M. smegmatis* coexpressing the indicated forms of *espA* and analyzed by SDS-PAGE under reducing conditions and Western blot analysis.

We sought to define unique functions of EspA that are independent of its role in the secretion of other ESX1 substrates. To do this we reasoned that EspA might participate in unique protein-protein interactions after secretion that could be targeted to disrupt EspA's post-secretory function. Indeed, when we analyzed culture filtrates from wildtype *Mtb* using SDS-PAGE in the absence of reducing agent, secreted EspA predominantly migrated with an apparent molecular mass of 80 kDa though smaller forms were detected (Figure 1A). Upon reduction, these forms of EspA resolved to a single species with an apparent molecular weight of 38 kDa, close to the predicted molecular weight of the monomer. As EspA contains a single cysteine at position 138, we hypothesized that after secretion, EspA dimerizes either with itself or with another protein via intermolecular disulfide bond formation. Of note, small amounts of the higher molecular weight forms of EspA were also detectable in the cell pellets (Figure 1A). We hypothesize that these represent secreted EspA that remains associated with the mycobacterial cell wall or perhaps is retained in the functional periplasmic space of the bacterium [30].

To identify the proteins that were disulfide-bonded to EspA, we affinity purified complexes associated with a C-terminally-tagged EspA allele, which we previously showed fully complements an EspA deletion mutant [27]. As a negative control we evaluated a strain carrying a deletion of the *espA* gene complemented with an empty vector in parallel. When affinity-purified proteins were resolved by SDS-PAGE and visualized by Coomassie staining, we identified bands specific to the EspA-his6 expressing strain only at 38kDa and 80 kDa. Western blot analysis indicated that both bands contained EspA (Figure 1B). Using tandem mass spectrometry, we identified only multiple unique peptides from EspA in both bands (Figure 1B, Table S1). A nonspecific 60 kDa band isolated from both strains was identified as *Mtb* GroEl1, a protein that contains a naturally occurring polyhistidine motif [31] and thus, would be expected to copurify. These data suggested that the 80 kDa species represents a homodimer of EspA which is covalently linked via an intermolecular disulfide bond.

To further test the model that EspA homodimerizes, we co-expressed EspA tagged with a FLAG epitope and EspA tagged with a Myc epitope in *M. smegmatis*. When heterologously expressed in *M. smegmatis*, EspA is found in both the 38 kDa and 80 kDa forms that are observed in *M. tuberculosis* (data not shown). As predicted, when EspA-Myc was affinity purified with an anti-Myc antibody from bacteria expressing EspA-Myc and EspA-FLAG, both the Myc- and FLAG- tagged forms of the protein were isolated, but EspA-FLAG was not isolated from the control strain which did not express EspA-Myc (Figure 1C).

Taken together, these data demonstrate that EspA homodimerizes and that a subset of these homodimers are covalently linked through intermolecular disulfide bond formation. Further analysis of secreted EspA suggested that the intermediate forms of EspA that migrate between the EspA dimer and monomer (Figure 1A) represent cleavage products of the EspA dimer (Figures S1A–C).

### Mutation of EspA cysteine 138 does not inhibit ESX1 secretion

Because disulfide bond formation occurs rarely in the reducing cytosolic environment [32], we reasoned that disulfide bond formation in the EspA dimer occurs after secretion and could, therefore, be targeted to disrupt EspA function but not interfere with ESX1 secretion. To test this prediction, we mutated the unique cysteine in EspA, at position 138, to alanine (*espA^C138A^*). We find that EspA is significantly more abundant when expressed in the context of the other genes in its operon, *espC* and *espD* (data not shown). To test the effect of the *espA^C138A^* mutation, we therefore generated an unmarked deletion of *espACD* and complemented this mutant with the wildtype *espACD* genes under the control of a tetracycline inducible promoter [33], a similar construct expressing *espA^C138A^CD* or an empty vector as a negative control.

**Figure 2:**
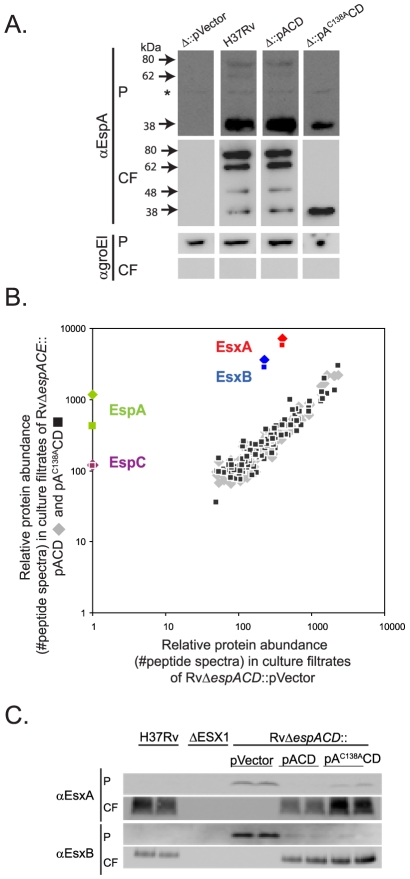
Inhibition of EspA disulfide bond formation does not abrogate ESX1 secretion. A. Western blot analysis of whole cell pellets (P) and short term culture filtrates (CF) of the indicated strains grown in N-salt media analyzed under non-reducing conditions. Arrows mark bands specific for EspA. The asterix marks a nonspecific background band. Results are representative of three independent experiments. Samples were assessed for GroEl1 to control for baterial autolysis. B. The relative abundance of proteins in culture filtrates collected in N-salt media from RvΔ*espACD* complemented with a control vector was compared to the abundance of proteins in RvΔ*espACD*::pACD (diamonds) or RvΔ*espACD*::pA^C138A^CD (squares). Protein abundance was determined by spectral count analysis. The lack of EspA and EspC in the culture filtrates from the deletion strain validates the quantitative method. Peptide counts are found in Table S2. Where no spectra were identified, an arbitrary value of 1 was assigned. Data were obtained from four biologically independent samples processed in duplicate and are representative of at least two fully independent experiments. C. Whole cell pellets (P) and short term culture filtrates (CF) from wildtype H37Rv, an ESX1 deletion mutant, and the RvΔ*espACD* complemented with the indicated constructs grown in Sauton's media. Samples were analyzed by SDS-PAGE under reducing conditions and Western blot analysis. Biologically independent duplicates were analyzed in each panel and these data are representative of at least 3 fully independent experiments.

We confirmed that when EspA was expressed in RvΔ*espACD*::pACD EspA was secreted and formed the same high molecular weight complexes that are observed in the culture filtrates of wildtype *Mtb* (Figure 2A). In contrast, EspA^C138A^ was secreted but did not form the SDS-stable dimer. Thus, mutation of the sole cysteine in EspA inhibits disulfide-bonding of the EspA dimer but does not inhibit EspA secretion.

To comprehensively determine whether *espA^C138A^* alters *Mtb* protein secretion we used quantitative tandem mass spectrometry to analyze the culture filtrate proteins of RvΔ*espACD*::pVector, RvΔ*espACD*::pACD and RvΔ*espACD*::pA^C138A^CD. To determine relative protein abundance, we made use of the fact that, using appropriate data acquisition parameters, the number of peptide spectra observed from a given protein directly reflects its overall abundance. Thus, we could estimate the relative abundance of each protein by quantifying the protein's spectral counts [34]–[35]. For robust quantitation, we focused on the 150 most abundant culture filtrate proteins, each of which was quantifiable by 75 or more spectra (Figure 2B and Table S2).

To validate the method, we assessed how the presence or absence of the *espACD* affected *Mtb* protein secretion. As previously shown [27], [36], we found that optimal EsxA and EsxB secretion requires the presence of the *espACD* operon (Figure 2C). By proteomic analysis, EsxA and EsxB secretion was ∼20 fold less efficient in the absence of *espACD* than presence of wildtype genes; however, EsxA and EsxB could still be identified in the culture filtrates of this strain (Figure 2B). By quantitative western blot analysis, we estimated that there was ∼100 fold less EsxA in the culture filtrates of *Mtb* lacking *espACD*, consistent with the proteomic data but suggesting that the quantitative dynamic range of the proteomic method is compressed. Interestingly, secreted isoforms of EsxA are found in the culture filtrates of the *espACD* deletion mutant () although we and others have not found them in culture filtrates from ESX1 mutants lacking core components of the ESX1 apparatus such as the FtsK-like ATPases, Rv3870 and Rv3871 (A. Garces and T. Ramsdel, unpublished data, and as previously shown in [6]), suggesting that optimal EsxA and EsxB secretion requires *espACD* but that residual EsxA and EsxB secretion occurs in the absence of these genes.

We then assessed the effect of inhibiting EspA disulfide bond formation on *Mtb* protein secretion. Loss of EspA disulfide bond formation did not substantially alter the global protein secretion profile of *Mtb* (Figure 2B & Table S2). Importantly, inhibition of EspA disulfide bond formation did not affect EsxA and EsxB secretion, which we confirmed by western blot analysis (Figure 2C). In *Mtb* expressing *espA^C138A^CD*, the proteomic analysis suggested that EspA secretion was intact though somewhat reduced relative to wildtype. EspC was also identified by mass spectrometry in the culture filtrates, as has been predicted by recently published studies in *M. marinum* [37], and the total secretion of EspC was not altered in bacteria expressing *espA^C138A^CD*. Thus, the proteomic data indicate that inhibition of EspA disulfide bond formation does not globally alter protein secretion in *Mtb* and is not required for ESX1 secretion of EsxA and EsxB.

### EspA disulfide bond formation is required for virulence of *Mtb*

**Figure 3:**
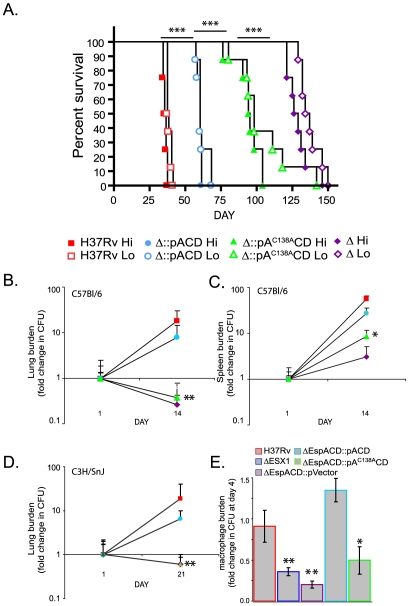
Inhibition of EspA disulfide bond formation significantly attenuates *Mtb* for virulence. A. SCID mice were infected intravenously with the indicated strains. RvΔ*espACD*::pVector is indicated as “Δ”. The goal “Hi” innoculum was 1×10^5^ organisms; the working stock of “Hi” innoculum was diluted 3 fold to obtain the “Lo” innoculum. Dosing, as confirmed by sacrificing 2 mice from the Hi group 24 hours after infection and plating for CFU, was H37Rv-6.2×10^4^, RvΔ*espACD*::pACD-9.5×10^4^, RvΔ*espACD*::pA^C138A^CD-6.6×10^4^, RvΔ*espACD*::pVector-22×10^4^. The differences in survival between groups of animals infected with the different strains were highly statistically significant by Chi Square test; differences between Hi groups are shown (***p<0.001). B–D. C57Bl/6 and C3H/HeSnJ mice were infected intravenously with the indicated strains. Organ burden is expressed as fold change from the organ burden at 24 hours. Data points represent mean+/− standard deviation of bacterial numbers from 4 mice/group. The organ burdens of H37Rv and RvΔ*espACD*::pACD were significantly greater than RvΔ*espACD*::pA^C138A^CD or RvΔ*espACD*::pVector by T-test as shown (*p<0.05, **p<0.01). E. Murine bone marrow derived macrophages were infected with the indicated strains of *Mtb*. Bacterial survival at day 4 relative to day 1 is plotted and represents the mean+/−standard deviation of 4 biologic replicates. The relative survival of the ESX1 deletion mutant, RvΔ*espACD*::pVector and RvΔ*espACD*::pA^C138A^CD was significantly less than that of H37Rv or RvΔ*espACD*::pACD by T-test as shown (*p<0.05, **p<0.01).

We hypothesized that inhibition of EspA disulfide bond formation would allow us to specifically identify aspects of ESX1 mediated virulence that require EspA function and dissociate them from those that require ESX1 secretion of EsxA and EsxB. To do this, we assessed the effect of the *espA^C138A^* mutation on the virulence of *Mtb*. In a SCID mouse model of infection, which was chosen in order to assess the virulence of the *Mtb* strains independent of the effects of adaptive immunity, animals infected with wildtype *Mtb* succumbed to infection after roughly 35 days (Figure 3A). The *espACD* deletion mutant was significantly attenuated for virulence; mice infected with this strain survived for an average of 127 days. Wildtype *espACD* significantly but incompletely complemented the deletion mutant for virulence. It is possible that the failure to fully complement the virulence defect is due to the fact that the *espACD* genes were ectopically expressed from an episomal vector under the control of an inducible promoter. Unlike the ESX1 locus, the *espACD* genes have been shown to be under the control of multiple regulators including EspR [26] and PhoPR [23]. Thus, it is not surprising that ectopic expression of the locus via an inducible promoter does not fully recapitulate the appropriate amount and timing of secretion during infection of an animal.

However, in comparison to the strain expressing wild type *espACD*, the strain of *Mtb* expressing *espA^C138A^CD* was significantly attenuated (Figure 3A). Mice infected with the strain expressing *espA^C138A^CD* survived 95 days on average, about 35 days longer than mice infected with bacteria expressing the wildtype *espACD*. We obtained very similar survival times in mice infected in parallel with a three fold dilution of each innocula, demonstrating that the differences in survival reflect marked differences in the virulence of the infecting strains rather than small differences in infecting doses (Figure 3A).

We also found that virulence depended critically on C138 of EspA in immunocompetent mice. In both C57Bl/6 and C3H/HeSnJ mice, whose MHC haplotype also allows us to simultaneously measure EsxA and EsxB specific T cell responses as described below, the *espACD* mutant complemented with *espA^C138A^CD* was attenuated to nearly the same extent as the deletion mutant complemented with an empty vector while complementation with wildtype *espACD* largely restored *Mtb* growth in lungs and spleen (Figures 3B–D). Loss of ESX1 has also been shown to attenuate *Mtb* for growth in macrophages [6]. We therefore assessed the ability of *Mtb* expressing *espA^C138A^CD* to survive in murine bone marrow derived macrophages. Like the ESX1 deletion mutant, *Mtb* lacking *espACD* or expressing *espA^C138A^CD* were attenuated for survival in macrophages (Figure 3E). Thus, we find that inhibition of EspA disulfide bond formation significantly attenuates the virulence of *Mtb* in animals and in macrophages despite apparently normal secretion of EsxA and EsxB.

### EspA disulfide bond formation is not required for ESX1-dependent activation of the innate and adaptive immune responses

**Figure 4:**
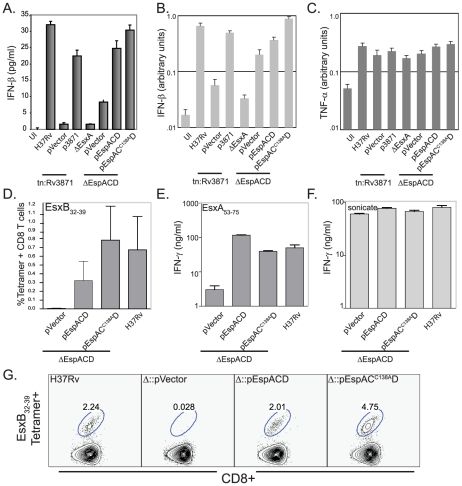
EspA disulfide bond formation is not required for innate or adaptive immune responses to *Mtb*. A. IFN-β secreted by RAW-264.7 cells infected with the indicated strains. IFN-β production was assessed by ELISA 24 hours after infection. Bars represent mean± standard deviation of three biologic replicates. Data are representative of at least 3 independent experiments. B, C. IFN-β and TNF-α expression by RAW-264.7 cells infected with the indicated strains 24 hours after infection. IFN-β and TNF-α expression was assessed by QT-PCR analysis and values were normalized to GAPDH expression. Bars represent mean± standard deviation of three biologic replicates and are representative of at least three independent experiments. D. Percent of CD8+ T cells which stain with H2-K^k^/EsxB_32–39_ tetramer. CD90+ T cells from the spleens of mice (n = 4) 3 weeks post-infection were analyzed individually and bar represent indicate mean values± standard deviation. There was a statistically significant difference in CD8+ T cell responses between mice infected with RvΔ*espACD*::pVector vs. H37Rv and RvΔ*espACD*::pA^C138A^CD (p<0.05 by T-test). E, F. Splenic CD90+ T cells isolated from infected mice (n = 4, pooled) 3 weeks post-infection with the indicated strains were restimulated *in vitro* with the CD4+ antigenic peptide, EsxA_53–75_ or H37Rv sonicate. Bars indicate means+/−standard deviations. The CD4+ responses to EsxA_53–75_ were significantly lower in animals infected with RvΔ*espACD*::pVector than in animals infected with the other strains (all p<0.01 by T-test). G. H2-K^k^/CFP10_32–39_ tetramer staining of CD90+ T cells pooled from the lungs of 4 mice 3 weeks post-infection with the given strains.

Strains expressing mutant EspA could be attenuated because they elicit different host responses or host damage. Because EsxA has been postulated to disrupt host cell membranes, we sought to determine whether *Mtb* expressing *espA^C138A^CD* retain the ability of perturb host cell membranes. To test this we took advantage the fact that ESX1 is required for the rapid induction of IFN-β transcription upon M. tuberculosis infection [7]. We have shown that maximal IFN-β expression depends on activation of the NOD2 pathway which is triggered by bacterial peptidoglycan in the host cell cytosol [13]. We therefore assessed the ability of *Mtb* expressing wildtype or mutant EspA to induce secretion of IFN-β after macrophage infection. As previously shown, wildtype *Mtb* activates IFN-β expression and secretion in an ESX1, *esxA* and *espA* dependent fashion Figure 4A–B) while loss of these virulence determinants did not affect induction of TNF-α (Figure 4C). Complementation of the *espACD* deletion mutant with *espA^C138A^CD* restored the ability of the cells to activate IFN-β production to the same extent as complementation with the wildtype genes. Thus, inhibition of EspA disulfide bond formation does not perturb the bacterium's ability to activate the cytosolic pattern receptors.

We extended these observations by assessing whether the *espA^C138A^* mutant's ability to stimulate the IFN-β response correlated with its ability to prime a CD8+ T cell response. EsxB is an important CD8+ T cell antigen in both mice and humans [38]–[39]. The path by which *Mtb* antigens reach the class I MHC processing pathway has not been well established. However, we have previously shown that ESX1 secretion is required in order to prime a CD8+ T cell response to EsxB [40]. We reasoned that the ESX1 substrates might strongly induce CD8+ T cell responses because they can gain access to the host cell cytosol and thus are readily sampled by the cytosolic class I MHC processing and presentation pathway. Consequently, we assessed the EsxB-specific CD8+ T cell response elicited by *Mtb* expressing *espA^C138A^CD*. We found a robust CD8+ T cell response to EsxB in the spleens and lungs of animals infected with RvΔ*espACD*::pA^C138A^CD (Figures 4D and 4G). These findings are consistent with the data showing this strain is also capable of secreting EsxB and inducing IFN-β production. As anticipated from previously published results [4], the CD4+ T cell response to EsxA is abrogated in the absence of *espACD* (Figure 4E). However, it is intact in animals infected with *espA^C138A^CD* (Figure 4E), providing evidence that EsxA is secreted *in vivo* as well as *in vitro* in the absence of EspA disulfide bond formation. T-cells from mice infected with the various mycobacterial mutants produced similar amounts of IFN-γ in response to mycobacterial whole cell lysate, indicating that the global T-cell response to *Mtb* was not affected by EspA disulfide bond formation (Figure 4F).

### Inhibition of EspA disulfide bond formation alters mycobacterial cell wall integrity

Spontaneous loss of ESX1 function during the laboratory evolution of both *M. bovis* BCG and H37Ra was associated with marked changes in colony morphology [21]–[22]. Complementation of BCG with a wildtype copy of the ESX1 genes resulted in colonies that again appeared similar to colonies of virulent *Mtb* [3]. More recent expression studies have also indicated that the *espACD* genes are highly transcriptionally regulated by cell wall stress [41]–[43], suggesting a link between these ESX1 substrates and cell wall structure. Based on these observations, we hypothesized that inhibition of EspA disulfide bond formation might alter the virulence of *Mtb* because it compromises the integrity of the cell wall.

**Figure 5:**
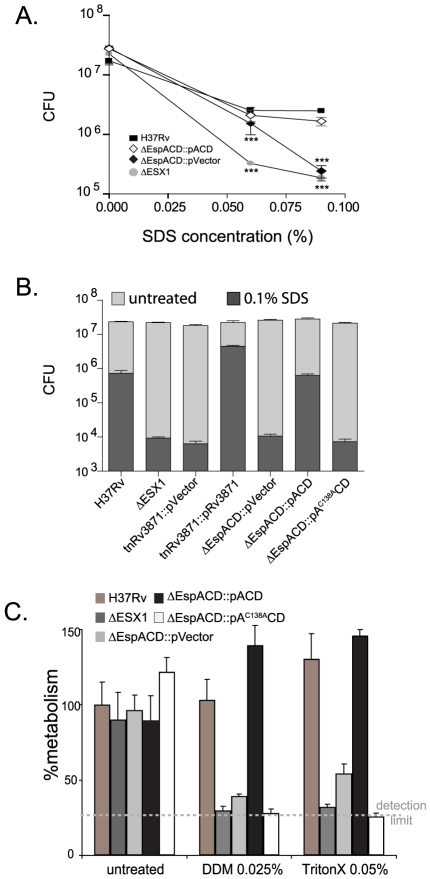
ESX1 and EspA activity are required for the ability of *Mtb* to survive cell wall stress. A. Bacterial survival after SDS stress. The indicated strains were exposed to increasing concentrations of SDS for 6 hours and then plated for CFU. Untreated bacteria were plated as a control and survival is shown. The mean+/−standard deviations of three independent biologic replicates are expressed. Strains lacking ESX1 and *espACD* were significantly more susceptible to SDS stress than H37Rv as assessed by T-test (***p<0.005). B. The indicated strains were exposed to treatment medium containing 0.1% SDS or control medium for 6 hours and then plated for CFU. Strains carrying tetracycline inducible vector and paired control strained were cultured in AT at 100 ng/ml overnight prior to and during SDS treatment. The mean+/−standard deviations of three independent biologic replicates are expressed. Data are representative of at least three fully independent experiments. Strains lacking ESX1 function, *espACD* and EspA disulfide bond formation were significantly more susceptible to SDS stress than H37Rv as determined by T-test (for these comparisons, all p<0.01). C. Bacterial survival after n-dodecyl beta-D-maltoside or TritonX-100 exposure. Bacterial metabolism as measured by Alamar blue after treatment with n-dodecyl beta-D-maltoside (DDM) orTritonX-100 expressed relative to the metabolism of untreated H37Rv. The mean+/−standard deviations of three independent biologic replicates are expressed. Strains lacking ESX1 function, *espACD* and EspA disulfide bond formation were significantly more susceptible to DDM and TritonX-100 as compared to H37Rv as determined by T-test (for these comparisons, all p<0.005).

Colony morphology is a subjective measure of cell wall structure and we have found it difficult to reproducibly and quantitatively score for ESX1 associated changes in colony morphology. Therefore, we sought more objective assays to assess cell wall integrity in our mutants. We found no evidence that loss of ESX1 or *espACD* altered bacterial resistance to reactive oxygen or nitrogen species (data not shown). However, we found that *Mtb* strains lacking the ESX1 locus, an FtsK-family ATPase in the ESX1 locus (Rv3871), or *espACD* were significantly more susceptible than wild type to a direct cell wall stress, SDS treatment (Figures 5A and 5B). Deletion of the ESX1 locus had a quantitatively greater effect on cell wall integrity than loss of *espACD*. The cell wall defect could be complemented by introduction of the wildtype genes (Figures 5A and 5B). We then assessed whether EspA disulfide bond formation was required for EspA's contribution to the cell wall integrity of *Mtb*. Strikingly, we found that bacteria expressing *espA^C138A^CD* show a similar susceptibility to SDS-induced stress as strains lacking the *espACD* locus entirely (Figure 5B). We tested other cell wall stressors and found that *Mtb* lacking ESX1, *espACD* or *Mtb* expressing *espA^C138A^CD* were also susceptible to other detergent stresses including n-dodecyl beta-D-maltoside and TritonX-100 (Figure 5C and Figure S3). Thus, ESX1 activity was required for the functional integrity of the mycobacterial cell wall and this effect requires EspA disulfide bond formation.

## Discussion

The ESX1 secretion system is critically required for the virulence of *Mtb* yet little is understood about its mechanism of action. One hypothesis is that EsxA is the primary mediator of ESX1-associated virulence, acting as a pore-forming molecule that allows the bacterium access to the host cell cytosol [11],[16],[44]. Alternatively, the ESX1 system might function like a type IV secretion system, secreting effector proteins directly into the host cell cytosol [45]. In both of these models, the ESX1-dependent stimulation of cytosolic immune pathways and CD8+ T cell responses has been used as evidence that the ESX1 system targets host cell membranes. These models of ESX1 function do not address the experimental observations that suggest that the ESX1 locus affects the composition of the mycobacterial cell wall.

In this work, we have dissociated ESX1 secretion and the effects of the ESX1 apparatus on the innate and adaptive immune systems from ESX1 dependent cell wall effects and virulence. Disruption of EspA disulfide bond formation does not perturb ESX1 secretion or ESX1 dependent interactions between *Mtb* and the innate and adaptive immune systems. It does, however, alter the functional integrity of the mycobacterial cell wall and dramatically attenuate the bacterium for virulence *in vivo*. These data suggest that ESX1 is required for *Mtb* to survive and cause disease *in vivo* at least in part because of its effects on the cell wall. Perturbation of the cell wall structure may attenuate the organism for growth *in vivo* because it broadly disrupts the bacterial interface with the host cell, undermining specific virulence functions, or because the organism is more susceptible to host antimicrobial defenses.

The most parsimonious explanation for our findings is that EspA acts directly on the mycobacterial cell wall. We and others have shown that EspA is secreted in standard mycobacterial growth media, which includes low concentrations of nonionic detergent. However, we have found that this protein remains associated with the cell wall when *Mtb was* grown in the absence of detergent (data not shown), in keeping with recently published microscopy data demonstrating that several ESX1 substrates are associated with the mycobacterial capsule in minimally disturbed bacterial cultures [46]. Thus, EspA could reasonably be engaged in modifying the cell wall and perhaps the capsule more specifically. For example, EspA may be directly required for the transport of cell wall components or may regulate the activity of other cell wall acting proteins. Alternatively, EspA could also have indirect effects that alter gene expression, although there is little evidence of ESX1 dependent changes in transcription [47].

We show that *Mtb* strains lacking ESX1 or EspA function have a marked defect in cell wall integrity as measured by detergent susceptibility. However, we have found that ESX1 function does not affect other measures of cell wall permeability or structure such as susceptibility to the hydrophobic antibiotic, rifampin, the cell wall acting antibiotics, isoniazid and meropenem, or lysozyme (data not shown). Our findings are consistent with studies of other cell wall mutants which have found that different mutants in cell wall biosynthesis have variable defects in permeability and susceptibility assays. In some cases, susceptibility can be easily predicted by gene function. For example, disrupting *ponA*, which acts on peptidoglycan, causes hypersusceptibility to lysozyme [48]. In many cases, however, the link between the genetic lesion and susceptibility to different cell wall stressors is not obvious [48]–[50], reflecting our limited insight into cell wall assembly in *Mtb*. In the case of ESX1, further biochemical analysis will be required to determine the specific cell wall defect caused by loss of activity.

Our model does not exclude the possibility that other ESX1 substrates, such as EsxA, have a direct activity on the macrophage as we find that activation of the host cytosolic surveillance systems occurs independently of EspA disulfide bond formation but requires ESX1 activity. The *espACD* deletion mutant is less virulent in SCID mice than *Mtb* lacking EspA disulfide bond formation, suggesting that isolated EsxA secretion may make an independent contribution to virulence in animals. However, EsxA, like the rest of the ESX1 locus, is highly conserved in both pathogenic and nonpathogenic gram positive bacteria [18], suggesting that this protein has an important biologic function in the bacterium that is a prerequisite for the virulence of *Mtb* but that it does not directly mediate virulence. Indeed, the data presented here suggest that the primary target of the ESX1 system is the bacterial cell wall.

## Materials and Methods

### Culture of Mtb and preparation of culture filtrates and cell lysates

*Mtb* and *M. smegmatis* strains were maintained as previously published [27], [51]. The EsxA deletion mutant and Rv3871 transposon mutant have been previously described [6]. For analysis of protein expression and secretion, bacteria from cultures normalized to the same growth phase were washed and resuspended in designated medium at an O.D.∼0.3 for 72 hours at 37°C. Where indicated, bacteria were cultured in N salt media (100 mM Bis/Tris HCl, 5 mM KCL, 7.5 mM (NH_4_)_2_SO_4_, 0.5 mM K_2_HSO_4_, 1 mM KH_2_PO_4_, 10 mM MgCl_2_, 38 mM glycerol, pH 7.0). N salt media is a minimal medium that allows titration of the divalent cation concentration which we have historically used when collecting samples for proteomic analysis [27]. Cell pellets and culture filtrates were collected and processed as described previously [27] except that culture filtrates were concentrated by precipitation with 10% trichloroacetic acid unless otherwise noted.

### Generation of mutant strains and complementing constructs

The genes encoding *espACD* were deleted from wildtype H37Rv through homologous recombination using a suicide vector approach. Deletion was confirmed by PCR analysis. As described in detail in Text S1, *espACD* was amplified from H37Rv genomic DNA and the *espA^C138A^* mutation was introduced via PCR mutagenesis and cloning of an internal gene fragment. The PCR products were recombined into a Gateway donor vector (Invitrogen, Carlsbad, CA) and transferred to an episomal expression vector (pTET) that was constructed to express genes under the control of a tetracycline inducible mycobacterial promoter [33]. All constructs were confirmed by sequencing. The constructs or an empty vector were transformed into RvΔ*espACD* to generate the designated strains. *Rv3871* was amplified (Forward primer: GGCTAAGAAGGAGATATACATATGACTGCTGAACCGGAAGTA; Reverse primer: CTTGTCGTCGTCGTCCTTGTAGTCACCGGCGCTTGGGGGTGC) and the PCR product was similarly recombined into pTET. This construct or an empty vector was transformed into the Rv3871 transposon insertion mutant. Gene expression was induced from the tetracycline inducible promoter with 100 ng/ml of anhydrotetracycline (AT) (Spectrum Chemicals, Gardena, CA) for 24 hours prior to beginning culture filtrate collections.

### Protein analysis

Samples were analyzed via SDS-PAGE and western blotting as previously published [27]. Where noted, samples were reduced with 10 mM dithiothreitol (DTT) for 30 minutes at 37°C prior to gel electrophoresis. Antibodies to EsxA, EsxB and EspA were published previously [27]. The antibody to poly-histidine (#NB600-318), which was used to detect GroEl1, was obtained from Novus Biologicals (Littleton, CO) as were antibodies to the Myc- and FLAG- epitopes. Antibodies were used according to the manufacturer's directions. In addition, where indicated, relevant gel slices were excised and analyzed by tandem mass spectrometry (MS/MS) as using published methods [52]–[54] and as described in Text S1. Affinity purification of EspA(his6) from RvΔespA::pEspA(6his) and EspA-FLAG and EspA-myc from *M. smegmatis* were performed as described in Text S1.

### Infection of mice and assessment of CD8+ T cell responses

BALB/c-SCID, C57Bl/6 and C3H/HeSnJ mice were purchased from Jackson Laboratory (Bar Harbor, ME). 24 hours prior to infection, mycobacterial strains were cultured overnight in media containing 100 ng/ml AT and mice were started on chow containing 2000 ppm tetracycline (Research Diets, New Brunswick, NJ). Mice were maintained on tet-chow through the course of the experiment. Mice were infected by intravenous tail vein injection and doses were confirmed by plating the innocula. At the indicated times, 4 mice/group were sacrificed and bacterial organ burden was determined by plating for CFU. Organs from C57Bl/6 mice were plated on medium in the presence and absence of hygromycin to assess for loss of the episomal plasmid over the course of the experiment. No significant vector loss was detected. Mice with organ burdens that differed by more than 5 fold from other animals in the group were considered missed injections and these data were discarded. CD8+ and CD4+ T cell responses were assessed as previously published [40]. In order to ensure that the infected mice had equivalent bacterial burdens at the time of T cell analysis, the infecting doses of RvΔ*espACD*::pVector and RvΔ*espACD*::pA^C138A^CD were ten fold higher than that of RvΔ*espACD*::pACD or H37Rv.

### Macrophage infections and cytokine responses

Bacterial strains were prepared and induced as described for murine infections. Murine bone marrow derived macrophages were prepared from C57Bl/6 mice according to previously published protocols [55]. After 7 days of culture, differentiated macrophages were frozen for future use. For infections, bone marrow derived macrophages were thawed and plated at a density of 2.5×10^4^ cells per well of a 96 well tissue culture treated plate and allowed to adhere overnight. Monolayers were washed, and infected with the indicated strains at an MOI of 10 to produce a final infection of roughly 1 bacterium/macrophage. Bacteria were spun onto the macrophage monolayer and infection was allowed to proceed for 3 hours. Monolayers were washed three times and fresh medium was added containing 100 ng/ml AT. At the indicated times after infection, monolayers were lysed with PBS-0.1%TritonX-100 and bacteria in the well were enumerated by plating serial dilutions.

For cytokine assays, RAW-264.7 macrophages were infected with the indicated strains at an MOI of 1 bacterium/macrophage as previously described [56]. At the indicated times, culture filtrates were removed and IFN-β secretion was assessed by ELISA for IFN-β (R&D Systems, Minneapolis, MN). In addition, RNA was isolated from infected macrophages as previously described [56]. 2 µg of RNA was transcribed into DNA using random hexamers with Superscript III reverse transcriptase (Invitrogen, Carlsbad, CA). Quantitative PCR assays were performed with TaqMan Gene Expression IFN-β, TNF-α and GAPDH assays (Applied Biosystems, Foster City, CA). For these assays, standard curves were generated using serial dilutions of pooled cDNA from macrophages 4 hours after LPS stimulation.

### Detergent susceptibility

*Mtb* strains were grown to early log phase (∼0.2 O.D. at 600nm) in Sauton's medium supplemented with 0.05% Tween-80. Strains complemented with tetracycline inducible constructs, pEmpty, pACD, pA^C138A^CD and pRv3871, were cultured overnight in Sauton's containing 100 ng/ml AT. Cells were pelleted, washed once and resuspended at a density of 1.2×10^8^ cells/ml in 7H9 medium containing the indicated concentrations of SDS, DDM (n-dodecyl β-D-maltoside) and Triton-X-100. DDM and Triton-X-100 were purchased from Sigma-Aldrich (St. Louis, MO). In studies of strains expressing the tetracycline inducible constructs the medium also contained AT at 100 ng/ml. Bacteria were incubated in SDS for 6h at 37°C with shaking, washed twice and then plated for CFU on 7H10 agar plates containing 10% OADC. For susceptibility to DDM and Triton-X-100, bacteria were incubated overnight with detergent on a shaker at 37°C, washed twice and resuspended in 7H9 media supplemented with 10% OADC and 0.05% Tween-80. Cultures were serially diluted (10-fold) onto 96 wells plate and their viability determined by adding 20 µl of 10× Alamar Blue dye (AbD Serotec, Raleigh, NC). After incubating at 37°C for 2 days, cells were fixed for 1 h with 2% paraformaldehyde and absorbance measured at 570 and 600 nm on a Versamax microplate reader using Softmax Pro version 5.3 (Molecular Devices, CA).

### Bioinformatics

Proteomics data analysis was performed as described in Text S1 according to published methods. Statistical analyses and graphing were otherwise performed with GraphPad Prism.

### Ethics

All animal experimentation was conducted following the National Institutes of Health guidelines for housing and care of laboratory animals and performed in accordance with Institutional regulations after protocol review and approval by the Harvard Medical Area Standing Committee on Animals.

## Supporting Information

Text S1 Text containing supplemental methods.
(0.04 MB DOC)

Table S1 Peptides identified in affinity purification of EspA-6his. Proteins were affinity purified with nickel agarose from whole cell lysates of RvΔEspA::pEspA(6his) or RvΔEspA::pVector. Purified proteins were resolved by SDS-PAGE and visualized by Coomassie staining. Visible bands and equivalent regions of the gel from the control strain were sent for analysis by tandem mass spectrometry. Proteins identified by two or more unique peptides are listed with the identifying peptides. Bands were analyzed LTQ-FT MS/MS (80 kDa and 60 kDa bands) or LCQ MS/MS (38 kDa band).
(0.05 MB DOC)

Table S2 Relative abundance of culture filtrate proteins from stains lacking EspACD, expressing wildtype EspACD or expressing EspA^C138A^CD. Abundance of culture filtrate proteins from strains RvΔEspACD::pVector, RvΔEspACD::pEspACD and RvΔEspACD::espA^C138A^CD as determined by quantitative tandem mass spectrometry. The number of independent spectra mapping to each protein is reported. Where redundant peptides map to multiple proteins, all matches are indicated. Where no spectra were identified, an arbitrary value of 1.0 was assigned. The relative ratio of protein abundance in RvΔEspACD::pEspACD vs. RvΔEspACD::espA^C138A^CD is reported. * indicates a statistically significant difference in peptide abundance between strains expressing *espACD* and *espA^C138A^CD* as determined by T-test with Benjamani and Hochberg correction for multiple testing (*p<0.01).
(0.31 MB DOC)

Figure S1 Mass spectrometric analysis of EspA isoforms. A. Protein composition of culture filtrates from the indicated strains as assessed by Coomassie staining of SDS-PAGE gel. Biologic duplicates are shown. Band indicated in red boxes are present in strains expressing the wildtype *espACD* operon but not in the *espACD* deletion mutant. These bands and matching regions from the control strain were analyzed via MS/MS. B. Quantitation of EspA associated spectra from each form of the protein. For each molecular weight species, the number (n) of spectra identified from EspA is indicated. The ratio represents the number of spectra which map to the carboxy terminus of EspA (residues 281–392) compared to the amino domain of EspA (residues 1–280). The gray bar represents the predicted ratio of carboxy/amino peptides in the EspA coding sequence assuming cleavage at residue 280. Significant differences from the predicted ratio were assessed by extended G-test (** p<0.01 and ***p<0.001). C. Model of EspA proteolysis and disulfide bond mediated dimerization based on peptide mapping and predicted molecular weights.
(0.78 MB EPS)

Figure S2 Quantitative western blot analysis of EsxA secretion in the presence and absence of *espACD*. A. Bacterial cell pellets and culture filtrates from normalized cultures of the indicated strains were analyzed by Western blot analysis. Culture filtrates from H37Rv were diluted as indicated. Total protein content in the culture filtrates of the two strains was equal by Coomassie staining (data not shown). B. EsxA abundance was determined by quantitative densitometry using the Alpha Innotech Imaging system and software (San Leadro, CA).
(0.63 MB EPS)

Figure S3 ESX1 and EspA activity are required for the ability of *Mtb* to survive cell wall stress. A–E. Bacterial survival after treatment with n-dodecyl beta-D-maltoside and TritonX-100. The indicated bacterial strains were left untreated or treated with the indicated detergent at the indicated concentration overnight. Cells were washed and then plated in 10 fold dilutions as indicated. After two days of recovery, bacterial metabolism was measured by Alamar blue and is taken as a measure of bacterial survival. All experiments were performed in triplicates and data is representative of three independent experiments.
(7.75 MB EPS)
