# Supplementary material for: EspA Acts as a Critical Mediator of ESX1-Dependent Virulence in Mycobacterium tuberculosis by Affecting Bacterial Cell Wall Integrity
Source: PLoS Pathog. 2010 Jun 24;6(6):e1000957. doi: 10.1371/journal.ppat.1000957 (PMC2891827; doi:10.1371/journal.ppat.1000957)
Supplement: Text S1 — Text containing supplemental methods. (0.04 MB DOC) [file ppat.1000957.s001.doc]

**Text S1**

**Generation of mutant strains and complementing constructs.**

To delete *espACD* from the *Mtb* genome via homologous recombination, 850 base pairs of upstream flanking sequence and 610 base pairs of downstream flanking sequence were joined via PCR stitching. This product was cloned into the NdeI site of the suicide vector pMP62 which carries hygromycin resistance and *sacB*. After transformation into H37Rv, selection was first performed on 7H10 agar containing hygromycin (50 μg/ml) and then on 3% sucrose.

The *espACD* cassette was generated through directional cloning.  *EspD*, including the *espC-espD* intergenic region, was amplified with a Pst1 containing forward primer and an Xba1 containing reverse primer and cloned into pBluescript (Agilent, Santa Clara, CA) to generate pBS-EspD*_int_*. A myc tag was added to the C-terminal of *espD* through a Spe1-BamHI digest using sites that were schemed into the reverse primer. *The espA-C* genes were PCR amplified together using a forward primer containing an EcoRV site and a reverse primer that contained a Pst1 site and an HA tag in frame with the C-terminus of *espC*. The PCR product was cloned into pBS-EspD*_int_*, creating pBS-*espACD*. The *espACD* cassette was then amplified through two rounds of PCR from pBS-*espACD,.* This added *att* sites for Gateway recombination of the product and a ribosomal binding site upstream from *espACD*. The *espACD* cassette was then recombined into pDONR 221 (Invitrogen, Carlsbad, CA) to generate pD-*espACD*. From pD-*espACD*, the cassette was then recombined into a modified Gateway expression vector, pTETsg that was engineered to express genes under the control of a tetracycline inducible mycobacterial promoter creating pACD.

**Site directed mutagenesis of EspA to generate vector expressing EspA^C138A^CD**

*EspA* was amplified in two fragments with primers that mutated cysteine 138 to an alanine. The 5’ fragment was amplified with forward primer (TAGTAAGAAGGAGATATACATATGAGCAGAGCGTTCATCATCGATC) and reverse primer (CATCGCGCCCGC**GGC**AAACGGCGCCTGGAAGGCGGC). The 3’ fragment was amplified with forward primer (CAGGCGCCGTTT**GCC**GCGGGCGCGATGGCCGTAGTG) and reverse primer (CAAGAAAGCTGGGTCTTAGACGACGTTTCGTACCAGCACC). The PCR products were joined via PCR stitching, using primers that also encoded *att* sites required for Gateway recombination. The product was then recombined into Gateway donor vector (pDONR 221), to create pD-*espA^C138A^*. A 607bp region spanning the C138A mutation was cut out of pD-*espA^C138A^* with Pml1 and SacI. The fragment containing the mutation was ligated into pD-*espACD*, which had also been digested with PmlI and SacI, to create pD-*espA^C138A^CD*. The *espA^C138A^CD* cassette was then recombined into the destination vector, pTETsg, to create pA^C138A^CD. All constructs were confirmed by sequencing.

The constructs expressing –myc and –FLAG tagged EspA were generated using standard approaches. The e*spA-myc* cassette has been described previously and was cloned from pALE4 [27] into pMV762 using standard methods. To improve EspA expression, the genes encoding EspC and EspD were amplified from *pACD* and cloned immediately 3’ of *esp-myc* in this construct. To generate pEspA-FLAG, the FLAG-epitope was added by PCR to *espA* and this construct was recombined into pTETsg using the Gateway system as described above. All constructs were confirmed by sequencing.

**Affinity purification of epitope tagged EspA**

RvΔespA::pEspA(6his), also known as pALE4, and RvΔespA::pJEB have been described previously [27]. Bacteria grown in 7H9 were lysed in protein extraction buffer (50 mM Tris-HCl pH7.5, 5 mM EDTA). SDS was added to a final concentration of SDS 2% and ascorbic acid to a final concentration of 1mM. Samples were boiled for 20 minutes and then removed from the BSL3. Samples were diluted 40 fold in buffer containing 0.5% Triton, 50 mM Tris-HCl pH8.0, 10 mM imidazole. 400μL of nickel agarose (Qiagen, Valencia, CA) was added to 200 mL of diluted sample and incubated with gentle agitation at 4°C overnight. The nickel agarose was collected by centrifugation. Bound proteins were eluted with elution buffer for native proteins as described by the manufacturer. Purified proteins were analyzed by SDS-PAGE and Western blot analysis for EspA, EsxA and EsxB. In parallel, an SDS-PAGE gel was stained with Coomassie reagent. Visible bands and equivalent regions in the control lane were excised and analyzed by tandem mass spectrometry as described below.

To confirm the EspA homodimer, EspA was expressed in *M. smegmatis* on heterologous vectors in which it was C-terminally fused to the –myc (pMVEspAmyc) or –FLAG epitopes (pEspAFlg) as described in the below. Gene expression was induced as described for *Mtb* strains. Protein complexes were isolated from whole cell lysates by immunoprecipitation with the Profound Myc IP/Co-IP kit (Thermo Scientific, Waltham, MA) according to the manufacturers’ instructions.

**Quantitative proteomics analysis**

For mass spectrometric analyses, samples were fractionated on a 10-20% Tricine gel (Invitrogen), 45mins at 100V. Gels were shrunk overnight by the addition of 50% ethanol and 7% acetic acid, and then allowed to swell for 1 hour in deionized water. Gels were stained with SimplyBlue Safe Stain (Invitrogen) for 45mins, imaged, and sliced horizontally into fragments of equal size based on the molecular weight markers.

In-gel reduction, alkylation and digestion was performed after destaining and rinsing the gel sections with two washes of 50% ethanol and 7% acetic acid, followed by two alternating washes with 50 mM ammonium bicarbonate and acetonitrile. After removal of the last acetonitrile wash, 100 μL of sequencing grade trypsin (Promega,Madison, WI) was added to each gel slice at a concentration of 6.6 μg/milliliter in 50 mM ammonium bicarbonate/10% acetonitrile. The gel slices were allowed to swell for 30 minutes on ice, after which the tubes were incubated at 37 degrees for 24 hours. Peptides were extracted with one wash of 100 μL of 50 mM ammonium bicarbonate/10% acetonitrile and one wash of 100 μL of 50% acetonitrile/0.1% formic acid. The extracts were pooled and frozen at -80 degrees, lyophilized to dryness and redissolved in 40 μL of 5% acetonitrile, 0.1% formic acid.

Samples were then loaded into a 96-well plate (AbGene) for mass spectrometry analysis on a Thermo Fisher Scientific Orbitrap XL, Thermo Fisher Scientific LTQ-FT or a Thermo Fisher Scientific LCQ Deca XP Plus as indicated (Thermo Fisher Scientific, Waltham, MA). For each run, 10 μL of each reconstituted sample was injected with a Famos Autosampler (Dionex, Sunnyvale, CA) and the separation was performed on a 75 μM x 20cm column packed with C_18_ Magic media (Michrom Biosciences, Auburn, CA) running at 250 nL/min provided from a Surveyor MS pump (Thermo Fisher Scientific) with a flow splitter with a gradient of 5-60% water 0.1% formic acid, acetonitrile 0.1% formic acid over the course of 120 minutes. Between each set of samples, standards from a mixture of 5 angiotensin peptides (Michrom Biosciences) were run for 2.5 hours to ascertain column performance and observe any potential carryover that might have occurred. The Orbitrap XL was run in a top eight configuration with one MS 60K resolution full scan and eight MS/MS scans. The LTQ-FT was run in a top nine configuration with one MS 200K resolution full scan and nine MS/MS scans and the LCQ Deca XP Plus was run in a top five configuration with one MS full scan and five MS/MS scans. Dynamic exclusion was set to 1 with a limit of 180 seconds with early expiration set to 2 full scans.

Peptide identifications were made using the database search algorithm, SEQUEST (Thermo Scientific, San Jose, CA). Spectra were searched against a composite database contained the predicted open reading frames annotated in the genome of H37Rv supplemented with those uniquely annotated in CDC1551. A reverse database strategy was employed to estimate false discovery rate (FDR) [52]. Peptides were filtered at a 1% FDR and clustered into proteins using an Occam’s approach, a strategy which produced protein identifications with probabilities of 0.98 or greater in parallel analyses using ProteinProphet (data not shown) [53]. We pooled spectral counts across gel slices and biologic replicates and then compared levels of protein expression between strains using an extended G-test [54].
