# Supplementary material for: EspA Acts as a Critical Mediator of ESX1-Dependent Virulence in Mycobacterium tuberculosis by Affecting Bacterial Cell Wall Integrity
Source: PLoS Pathog. 2010 Jun 24;6(6):e1000957. doi: 10.1371/journal.ppat.1000957 (PMC2891827; doi:10.1371/journal.ppat.1000957)
Supplement: Table S1 — Peptides identified in affinity purification of EspA-6his. Proteins were affinity purified with nickel agarose from whole cell lysates of RvΔEspA::pEspA(6his) or RvΔEspA::pVector. Purified proteins were resolved by SDS-PAGE and visualized by Coomassie staining. Visible bands and equivalent regions of the gel from the control strain were sent for analysis by tandem mass spectrometry. Proteins identified by two or more unique peptides are listed with the identifying peptides. Bands were analyzed LTQ-FT MS/MS (80 kDa and 60 kDa bands) or LCQ MS/MS (38 kDa band). (0.05 MB DOC) [file ppat.1000957.s002.doc]

| **MW (kDa)**  **Supplemental Table 1** | **RvΔEspA::pEspA(6His)** | **RvΔEspA::empty vector** |
| --- | --- | --- |
|  |  |  |
| **80** | **EspA** | **none** |
|  | K.YSEGAAAGTEDAER.A |  |
|  | R.APVEADAGGGQKVLVR.N |  |
|  | R.QLISLIHDQANAVQTTR.D |  |
|  | K.YSEGAAAGTEDAERAPVEADAGGGQK.V |  |
|  | R.ADGPVGAAAEQVGGQSQLVSAQGSQGM*GGPVGM*GGM*HPSSGASKGTTTK.K |  |
| **60** | **GroEl1** | **GroEl1** |
|  | K.LAGGVAVIK.V | K.LAGGVAVIK.V |
|  | R.KALTELR.A | R.KALTELR.A |
|  | K.VGAATETALK.E | K.ESVEDAVAAAK.A |
|  | K.LIEYDETAR.R | R.EVGLEVLGSAR.R |
|  | R.EVGLEVLGSAR.R | K.TGIAQVATVSSR.D |
|  | K.TGIAQVATVSSR.D | K.VGAATETALKER.K |
|  | R.KESVEDAVAAAK.A | K.ISSLPDLLPLLEK.V |
|  | K.VGAATETALKER.K | K.ERKESVEDAVAAAK.A |
|  | K.AVAVKGPYFGDR.R | R.AM*EVGM*DKLADTVR.V |
|  | R.LVAAGVNPIALGVGIGK.A | R.M*VLTTETVVVDKPAK.A |
|  | R.AM*EVGM*DKLADTVR.V | K.AADAVSEALLASATPVSGK.T |
|  | K.AADAVSEALLASATPVSGK.T | K.DDTVIVDGGGTAEAVANR.A |
|  | K.DDTVIVDGGGTAEAVANR.A | R.EIELEDPFEDLGAQLVK.S |
|  | K.AAVEEGIVPGGGASLIHQAR.K | R.VVVSKDDTVIVDGGGTAEAVANR.A |
|  | R.VVVSKDDTVIVDGGGTAEAVANR.A | K.TNDVAGDGTTTATILAQALIKGGLR.L |
|  | K.TGIAQVATVSSRDEQIGDLVGEAM*SK.V | K.AFLEDLAVVTGGQVVNPDAGM*VLR.E |
|  |  | K.TGIAQVATVSSRDEQIGDLVGEAM*SK.V |
| **38** | **EspA** | **none** |
|  | K.KYSEGAAAGTEDAERAPVEADAGGGQK.V |  |
|  | K.YSEGAAAGTEDAERAPVEADAGGGQK.V |  |
