# Supplementary material for: EspA Acts as a Critical Mediator of ESX1-Dependent Virulence in Mycobacterium tuberculosis by Affecting Bacterial Cell Wall Integrity
Source: PLoS Pathog. 2010 Jun 24;6(6):e1000957. doi: 10.1371/journal.ppat.1000957 (PMC2891827; doi:10.1371/journal.ppat.1000957)
Supplement: Table S2 — Relative abundance of culture filtrate proteins from stains lacking EspACD, expressing wildtype EspACD or expressing EspAC138ACD. Abundance of culture filtrate proteins from strains RvΔEspACD::pVector, RvΔEspACD::pEspACD and RvΔEspACD::espAC138ACD as determined by quantitative tandem mass spectrometry. The number of independent spectra mapping to each protein is reported. Where redundant peptides map to multiple proteins, all matches are indicated. Where no spectra were identified, an arbitrary value of 1.0 was assigned. The relative ratio of protein abundance in RvΔEspACD::pEspACD vs. RvΔEspACD::espAC138ACD is reported. * indicates a statistically significant difference in peptide abundance between strains expressing espACD and espAC138ACD as determined by T-test with Benjamani and Hochberg correction for multiple testing (*p<0.01). (0.31 MB DOC) [file ppat.1000957.s003.doc]

| ***gene*** | ***Vector*** | ***EspACD*** | ***EspAC183ACD*** | ***ratio*** |  |
| --- | --- | --- | --- | --- | --- |
| Rv0002 | 142 | 130 | 135 | 1.0 | * |
| Rv0005  **Supplemental Table 2** | 78 | 142 | 136 | 1.0 |  |
| Rv0006 | 189 | 215 | 180 | 1.2 |  |
| Rv0009 | 170 | 95 | 165 | 0.6 | * |
| Rv0040c | 133 | 149 | 113 | 1.3 |  |
| Rv0041 | 144 | 131 | 147 | 0.9 | * |
| Rv0054 | 80 | 75 | 97 | 0.8 | * |
| Rv0062 | 223 | 165 | 146 | 1.1 |  |
| Rv0066c | 229 | 230 | 206 | 1.1 |  |
| Rv0125 | 301 | 272 | 231 | 1.2 |  |
| Rv0129c | 583 | 568 | 493 | 1.2 |  |
| Rv0148 | 79 | 66 | 80 | 0.8 |  |
| Rv0179c | 288 | 232 | 184 | 1.3 |  |
| Rv0211 | 207 | 261 | 210 | 1.2 |  |
| Rv0242c | 331 | 353 | 429 | 0.8 | * |
| Rv0248c | 238 | 307 | 376 | 0.8 | * |
| Rv0270 | 172 | 190 | 181 | 1.0 |  |
| Rv0285 | 154 | 94 | 86 | 1.1 |  |
| Rv0287 | 523 | 423 | 439 | 1.0 | * |
| Rv0288 | 50 | 97 | 36 | 2.7 |  |
| Rv0309 | 262 | 295 | 256 | 1.2 |  |
| Rv0315 | 150 | 194 | 198 | 1.0 |  |
| Rv0350 | 1517 | 1678 | 1968 | 0.9 | * |
| Rv0363c | 172 | 148 | 233 | 0.6 | * |
| Rv0384c | 622 | 551 | 726 | 0.8 | * |
| Rv0440 | 2299 | 2210 | 2957 | 0.7 | * |
| Rv0462 | 316 | 274 | 390 | 0.7 | * |
| Rv0467 | 158 | 132 | 126 | 1.0 |  |
| Rv0468 | 159 | 136 | 175 | 0.8 | * |
| Rv0469 | 51 | 87 | 98 | 0.9 | * |
| Rv0503c | 84 | 102 | 98 | 1.0 |  |
| Rv0632c | 154 | 140 | 177 | 0.8 | * |
| Rv0639 | 152 | 128 | 124 | 1.0 |  |
| Rv0642c | 134 | 102 | 119 | 0.9 | * |
| Rv0652 | 131 | 112 | 164 | 0.7 | * |
| Rv0667 | 664 | 577 | 521 | 1.1 |  |
| Rv0668 | 934 | 886 | 764 | 1.2 |  |
| Rv0684 | 250 | 316 | 278 | 1.1 |  |
| Rv0685 | 956 | 832 | 942 | 0.9 | * |
| Rv0815c | 292 | 170 | 281 | 0.6 | * |
| Rv0824c | 104 | 90 | 90 | 1.0 |  |
| Rv0831c | 213 | 215 | 252 | 0.9 | * |
| Rv0860 | 436 | 419 | 507 | 0.8 | * |
| Rv0873 | 114 | 220 | 221 | 1.0 |  |
| Rv0884c | 87 | 76 | 101 | 0.8 | * |
| Rv0888 | 107 | 143 | 74 | 1.9 | * |
| Rv0896 | 398 | 336 | 437 | 0.8 | * |
| Rv0905 | 113 | 88 | 143 | 0.6 | * |
| Rv0934 | 136 | 127 | 165 | 0.8 | * |
| Rv0951 | 175 | 153 | 154 | 1.0 | * |
| Rv0952 | 154 | 139 | 153 | 0.9 | * |
| Rv1023 | 193 | 184 | 189 | 1.0 |  |
| Rv1038c | 2101 | 2167 | 1361 | 1.6 | * |
| Rv1074c | 163 | 139 | 160 | 0.9 | * |
| Rv1093 | 135 | 118 | 147 | 0.8 | * |
| Rv1096 | 70 | 105 | 102 | 1.0 |  |
| Rv1098c | 182 | 176 | 190 | 0.9 | * |
| Rv1133c | 1393 | 1172 | 1268 | 0.9 | * |
| Rv1196 | 667 | 793 | 492 | 1.6 | * |
| Rv1197 | 2040 | 2166 | 1359 | 1.6 | * |
| Rv1198 | 1361 | 1533 | 939 | 1.6 | * |
| Rv1240 | 215 | 200 | 253 | 0.8 | * |
| Rv1308 | 310 | 326 | 333 | 1.0 |  |
| Rv1309 | 58 | 89 | 96 | 0.9 | * |
| Rv1310 | 307 | 282 | 319 | 0.9 | * |
| Rv1323 | 83 | 85 | 98 | 0.9 | * |
| Rv1328 | 320 | 249 | 286 | 0.9 | * |
| Rv1386 | 158 | 173 | 197 | 0.9 | * |
| Rv1387 | 438 | 458 | 451 | 1.0 |  |
| Rv1392 | 251 | 219 | 226 | 1.0 | * |
| Rv1436 | 208 | 220 | 233 | 0.9 |  |
| Rv1448c | 138 | 126 | 136 | 0.9 |  |
| Rv1449c | 119 | 252 | 256 | 1.0 |  |
| Rv1463 | 63 | 72 | 93 | 0.8 | * |
| Rv1475c | 684 | 575 | 672 | 0.9 | * |
| Rv1477 | 271 | 273 | 251 | 1.1 |  |
| Rv1479 | 105 | 84 | 98 | 0.9 |  |
| Rv1566c | 152 | 124 | 125 | 1.0 |  |
| Rv1617 | 141 | 142 | 150 | 0.9 |  |
| Rv1629 | 130 | 114 | 110 | 1.0 |  |
| Rv1630 | 396 | 469 | 432 | 1.1 |  |
| Rv1636 | 87 | 92 | 114 | 0.8 | * |
| Rv1754c | 267 | 309 | 252 | 1.2 |  |
| Rv1771 | 80 | 78 | 109 | 0.7 | * |
| Rv1793 | 1433 | 1632 | 1033 | 1.6 | * |
| Rv1837c | 547 | 535 | 717 | 0.7 | * |
| Rv1860 | 117 | 137 | 99 | 1.4 |  |
| Rv1886c | 431 | 429 | 414 | 1.0 |  |
| Rv1908c | 923 | 612 | 679 | 0.9 | * |
| Rv1926c | 318 | 314 | 289 | 1.1 |  |
| Rv1980c | 494 | 412 | 427 | 1.0 |  |
| Rv1984c | 114 | 100 | 96 | 1.0 |  |
| Rv2202c | 100 | 103 | 120 | 0.9 | * |
| Rv2215 | 233 | 347 | 391 | 0.9 | * |
| Rv2220 | 309 | 271 | 330 | 0.8 | * |
| Rv2241 | 279 | 247 | 275 | 0.9 | * |
| Rv2244 | 253 | 228 | 273 | 0.8 | * |
| Rv2245 | 98 | 95 | 117 | 0.8 | * |
| Rv2299c | 254 | 304 | 355 | 0.9 | * |
| Rv2301 | 176 | 158 | 187 | 0.8 | * |
| Rv2346c | 1630 | 2232 | 1202 | 1.9 | * |
| Rv2352c | 154 | 224 | 91 | 2.5 | * |
| Rv2391 | 104 | 101 | 90 | 1.1 |  |
| Rv2427c | 105 | 70 | 100 | 0.7 | * |
| Rv2430c | 125 | 106 | 133 | 0.8 | * |
| Rv2448c | 123 | 135 | 162 | 0.8 | * |
| Rv2467 | 246 | 201 | 236 | 0.9 | * |
| Rv2476c | 322 | 274 | 205 | 1.3 |  |
| Rv2525c | 154 | 219 | 240 | 0.9 | * |
| Rv2555c | 163 | 114 | 114 | 1.0 |  |
| Rv2783c | 442 | 333 | 321 | 1.0 |  |
| Rv2831 | 81 | 81 | 118 | 0.7 | * |
| Rv2878c | 210 | 175 | 163 | 1.1 |  |
| Rv2889c | 283 | 250 | 317 | 0.8 | * |
| Rv2911 | 118 | 109 | 95 | 1.1 |  |
| Rv2940c | 715 | 513 | 433 | 1.2 |  |
| Rv2971 | 53 | 67 | 95 | 0.7 | * |
| Rv2996c | 192 | 173 | 156 | 1.1 |  |
| Rv3028c | 387 | 272 | 317 | 0.9 | * |
| Rv3029c | 142 | 117 | 139 | 0.8 | * |
| Rv3036c | 89 | 79 | 77 | 1.0 |  |
| Rv3136 | 75 | 92 | 120 | 0.8 | * |
| Rv3224 | 147 | 132 | 193 | 0.7 | * |
| Rv3246c | 131 | 80 | 157 | 0.5 | * |
| Rv3248c | 208 | 171 | 249 | 0.7 | * |
| Rv3274c | 145 | 138 | 180 | 0.8 | * |
| Rv3280 | 158 | 151 | 158 | 1.0 |  |
| Rv3285 | 266 | 287 | 283 | 1.0 |  |
| Rv3389c | 115 | 93 | 111 | 0.8 | * |
| Rv3417c | 594 | 572 | 620 | 0.9 | * |
| Rv3418c | 506 | 352 | 984 | 0.4 | * |
| Rv3457c | 445 | 404 | 418 | 1.0 | * |
| Rv3477 | 294 | 263 | 176 | 1.5 |  |
| Rv3478 | 625 | 679 | 363 | 1.9 | * |
| Rv3596c | 176 | 144 | 93 | 1.5 |  |
| Rv3615c | 1 | 120 | 117 | 1.0 |  |
| Rv3616c | 1 | 1174 | 428 | 2.7 | * |
| Rv3646c | 132 | 141 | 183 | 0.8 | * |
| Rv3710 | 53 | 135 | 147 | 0.9 |  |
| Rv3722c | 111 | 90 | 119 | 0.8 | * |
| Rv3774 | 291 | 152 | 178 | 0.9 | * |
| Rv3800c | 476 | 372 | 313 | 1.2 |  |
| Rv3803c | 288 | 268 | 256 | 1.0 |  |
| Rv3804c | 637 | 515 | 497 | 1.0 |  |
| Rv3841 | 280 | 251 | 296 | 0.8 | * |
| Rv3846 | 128 | 106 | 160 | 0.7 | * |
| Rv3865 | 63 | 98 | 103 | 1.0 |  |
| Rv3874 | 225 | 3570 | 2797 | 1.3 |  |
| Rv3875 | 397 | 7233 | 5843 | 1.2 |  |
